# Supplementary material for: Identification of photoperiod-induced specific miRNAs in the adrenal glands of Sunite sheep (Ovis aries)
Source: Front Vet Sci. 2022 Jul 22;9:888207. doi: 10.3389/fvets.2022.888207 (PMC9354845; doi:10.3389/fvets.2022.888207)
Supplement: Supplementary file 1 [file Data_Sheet_1.ZIP › Supplementary file/miRDeep2 file.zip!/miRDeep2 file/Novel_Structure/LP42A2/Novel_519.pdf]

Provisional ID	: NC_019470.2_22816
Score total	: 1.6
Score for star read(s)	: -1.3
Score for read counts	: 0
Score for mfe	: 1.3
Score for randfold	: 1.6
Score for cons. seed	:
Total read count	: 24
Mature read count	: 23
Loop read count	: 1
Star read count	: 0

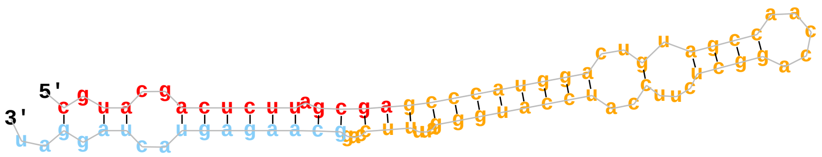

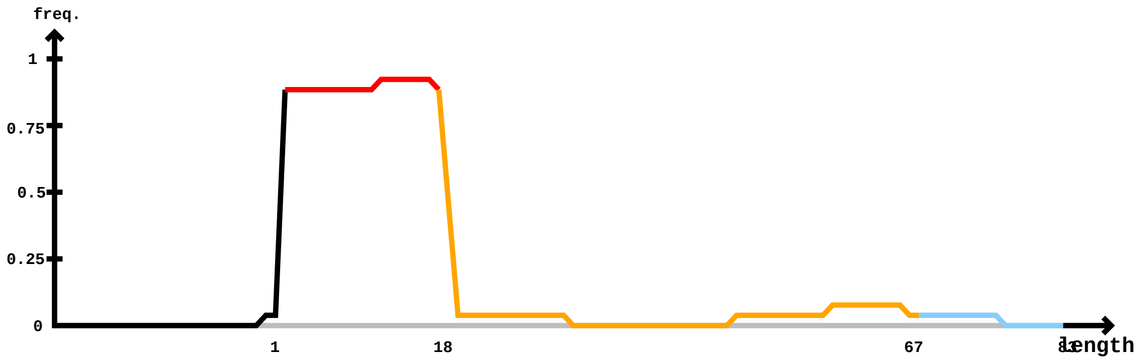

Mature   Star

```
5'- ugaaagugaagucgcucagucacguacgacucuuagcgagcccauggacuguagccaaccaggcucuuccauccaugggauuuuccaggcaagaguacuaggaugacu   -3'    exp
    ....((((....)))).((((((.((..((((((.(((((((((((((..(.((((.....))))...)..))))))))...)))...))))))))..)).).)))))    reads    mm    sample
    ....................cacguacgacucuuagcg......................................................................    1        0     seq
    ......................cguacgacucuuagcga.....................................................................    22       0     seq
    ................................uuagcgaUcccauggacugu........................................................    1        1     seq
    .....................................................................cauccaugggauuuucca.....................    1        0     seq
    ...............................................................................auuuuccaAgcaagagua...........    1        1     seq
```
